# Framing the Issues: Moral Distress in Health Care

**DOI:** 10.1007/s10730-012-9176-y

**Published:** 2012-03-25

**Authors:** Bernadette M. Pauly, Colleen Varcoe, Jan Storch

**Affiliations:** 1 School of Nursing, University of Victoria, Box 1700, STN CSC, Victoria, BC USA; 2 Centre for Addictions Research of BC, University of Victoria, Box 1700, STN CSC, Victoria, BC USA; 3 University of British Columbia, Vancouver, Canada; 4 University of Victoria, Victoria, BC USA

**Keywords:** Moral distress, Healthcare, Ethical practice, Recruitment, Retention

## Abstract

Moral distress in health care has been identified as a growing concern and a focus of research in nursing and health care for almost three decades. Researchers and theorists have argued that moral distress has both short and long-term consequences. Moral distress has implications for satisfaction, recruitment and retention of health care providers and implications for the delivery of safe and competent quality patient care. In over a decade of research on ethical practice, registered nurses and other health care practitioners have repeatedly identified moral distress as a concern and called for action. However, research and action on moral distress has been constrained by lack of conceptual clarity and theoretical confusion as to the meaning and underpinnings of moral distress. To further examine these issues and foster action on moral distress, three members of the University of Victoria/University of British Columbia (UVIC/UVIC) nursing ethics research team initiated the development and delivery of a multi-faceted and interdisciplinary symposium on Moral Distress with international experts, researchers, and practitioners. The goal of the symposium was to develop an agenda for action on moral distress in health care. We sought to develop a plan of action that would encompass recommendations for education, practice, research and policy. The papers in this special issue of *HEC Forum* arose from that symposium. In this first paper, we provide an introduction to moral distress; make explicit some of the challenges associated with theoretical and conceptual constructions of moral distress; and discuss the barriers to the development of research, education, and policy that could, if addressed, foster action on moral distress in health care practice. The following three papers were written by key international experts on moral distress, who explore in-depth the issues in three arenas: education, practice, research. In the fifth and last paper in the series, we highlight key insights from the symposium and the papers in the series, propose to redefine moral distress, and outline directions for an agenda for action on moral distress in health care.

Researchers have shown that moral distress is a wide spread problem for health care providers including nurses, pharmacists, social workers, physicians, and health care managers in a wide range of acute and community health care settings (Brazil et al. 2010; Hamric 2010; Hamric and Blackhall 2007; Kälvemark et al. 2004; Pauly et al. 2009; Ulrich et al. 2007; Wilkinson 1988). Moral distress is defined variously in different studies. While there are many sources of stress in health care work, moral distress is specifically associated with the ethical dimensions of practice and concerns related to difficulties navigating practice while upholding professional values, responsibilities and duties (Epstein and Hamric 2009; Hardingham 2004; Kälvemark et al. 2004; Kälvemark Sporrong et al. 2006). Moral distress, regardless of being understood differently in different studies, has been shown to have negative consequences, contributing to emotional distress (e.g., anger and frustration), withdrawal of self from patients, unsafe or poor quality of patient care, decreasing job satisfaction and even attrition in nursing (Cavaliere et al. 2010; Corley et al. 2005; Gutierrez 2005; Wilkinson 1988).

Hamric and colleagues have suggested that when nurses experience moral distress they may respond in at least one of three ways (Epstein and Hamric 2009; Hamric 2010). Nurses may withdraw from ethically challenging situations; change positions; and/or continue to raise objections and voice concerns about situations. Others have shown that the challenges many nurses face in fulfilling their commitments to people receiving care impact their decisions to stay in nursing or leave the profession (O`Brien-Pallas et al. 1994; Shamian et al. 2002; Torgerson 2007). Outcomes of moral distress, such as decisions to leave nursing, have been cause for increasing attention and concern among health care leaders, with some arguing that systemic changes are needed as part of a response to apparently rising levels of moral distress in health care (Hamric 2010; Kälvemark et al. 2004).

Research on moral distress has been plagued by a lack of conceptual and theoretical clarity that in turn, has hampered action on moral distress in education, policy and practice. Writing in an Irish context, McCarthy and Deady (2008) suggest that moral distress is a useful concept in nursing but that nursing discourse on moral distress has been confusing and counterproductive. They raise two concerns: (1) research on moral distress has lacked conceptual clarity and (2) research on moral distress “perpetuates the dominant or meta-narratives about the identity of professional nursing that, we think, ought to be challenged” (p. 258). McCarthy and Deady suggest that the concept of moral distress has perpetuated the meta-narrative of the moral suffering of nurses and the nurse as a victim. They observe that moral distress has been used with various understandings of preconditions for the development of such distress and conflated with psychological distress. These authors argue that we need a more critical stance towards moral distress and additional explorations of moral distress in relation to the ethical dimensions of practice. They suggest the need for an overhaul of the concept of moral distress including examination of philosophical perspectives informing moral decision making and accompanying emotional responses. Based on our own qualitative and quantitative work in relation to moral distress and ethical climates we heartily concur (Pauly et al. 2009; Rodney et al. 2002; Storch et al. 2002; Varcoe et al. 2004). Our research has highlighted concerns about moral distress in everyday nursing practice, and the limitations of the current state of knowledge development in relation to moral distress.

In response to these emerging concerns, we convened a two day symposium on moral distress in health care (Pauly et al. 2010). We invited key experts in the field to act as provocateurs in each of the following areas for action: education, practice, policy and research. We structured the sessions to be interactive and invited commentary in order to further dialogue on moral distress and promote the development of an agenda for action on moral distress. Over 75 people attended including international participants and representatives from a broad range of disciplines. The work was supported by two Canadian Institute of Health Research (CIHR) grants: a Meeting, Planning and Dissemination Grant, and a seed grant on Health Policy Ethics. The series of papers in this special issue of *HEC Forum* arose from this symposium.

The purpose of this initial paper in the series is to examine the issues related to lack of conceptual and theoretical clarity in work on moral distress in health care. Specially, we raised issues about the framing of moral distress in relation to individual and structural factors. In this paper, we provide an introduction to the concept of moral distress and make explicit some of the challenges associated with theoretical constructions of moral distress and the way in which these challenges have hampered action on moral distress in policy, research, practice and education. We argue that conceptual and theoretical clarity are key to development of an agenda on moral distress to guide policy, practice, education and research. This is particularly relevant to developing interventions, teaching practitioners, and guiding measurement and intervention research.

## Conceptual and Theoretical Tensions

Understandings of moral distress differ by the extent to which the problem is located in individual and/or structural factors. Jameton (1984) first coined the term moral distress to capture the inability of nurses to act on what they believe is the right thing to do because of institutional constraints. Jameton’s definition has been widely used in nursing and health care and emphasizes institutional and external constraints on the ability of nurses to practice ethically. His definition suggests that the constraints on the moral agency of nurses are beyond the control of individuals or located in the various institutions or structures that shape nurses’ work. As a follow-up to his initial work, Jameton suggested that individuals may experience initial and reactive moral distress (Jameton 1993). Reactive moral distress is a response to not acting on initial frustration experienced when encountering institutional obstacles and value conflicts.

Kälvemark et al. (2004) argued that Jameton’s definition assumes that if nurses take action they will not experience moral distress. Based on their research with nurses, pharmacists, physicians and other clinical staff, these authors found that health care providers reported moral distress when they had to make difficult choices between following rules or following their conscience. Thus, they acted and made choices, but still experienced distress related to ethical dimensions of practice. Based on their research, Kälvemark et al. revised Jameton’s definition of moral distress to “traditional negative stress symptoms that occur due to situations that involve ethical dimensions and where the health care provider feels she/he is not able to preserve all interests and values at stake” (pp. 1082–1083). This revised definition brings a clearer focus on both individual and structural factors in defining moral distress.

Webster and Baylis (2000) highlighted more specifically the individual and “perceived constraints”, including personal failings that prevent individuals from acting in ways that compromise their personal integrity. Of note, they delineated the negative effects of unresolved moral distress (moral residue) that can linger and impact practice overtime. Epstein and Hamric (2009) propose that moral residue (unresolved and reactive moral distress) is more common than has been previously recognized and likely exacerbated by problematic unit, team or institutional conditions. Webster and Baylis also observed that resolution of moral distress is a possible means of strengthening ethical practice in the future. In a review of moral distress literature, Hanna (2004) suggested that successful management of moral distress can be an opportunity for personal transformation and growth. However, we would add that successful management of moral distress by the individual requires attention to the broader structural conditions in which moral distress arises and can be resolved. For example, organizational and institutional supports are required to provide opportunities for resolution and attention to conditions are needed to prevent moral distress. Clearly, the relationships amongst individual experiences of moral distress, structural determinants of moral distress, moral agency and ethical action are not well understood.

As described above, much of our understanding of moral distress is from reading of North American literature. Internationally, there is a body of work on moral distress, particularly emanating from the Scandinavian countries, that uses different terminologies to refer to what appear to be similar concepts (Glasberg et al. 2006; Lützén et al. 2000, 2003, 2006). Lützén et al. (2003) state “moral stress is experienced when nurses are aware of what ethical principles are at stake in a specific situation and external factors prevent them from making a decision that would reduce the conflict between the contradicting principles” (p. 314). Further, Lützén et al. note that moral stress can be viewed as similar to moral distress in that the moral component is present in both concepts but that moral stress captures physiological dimensions not usually associated with moral distress. While some of the differences might relate to translation alone, a greater understanding and integration of North American, Scandinavian and European concepts could be helpful to enhancing conceptual clarity of moral distress.

The proliferation of writing and research on moral distress suggests that the phenomenon is an international concern, but there has been little work to assess the extent and degree to which this is the case or to examine structural conditions that give rise to different experiences of moral distress among health care providers. In the next article in this series, Lützén engages with and extends discussion on these and other issues. Lützén argues for development of a conceptual model as an alternative approach to guiding research on moral distress rather than embracing a single definition. In the next paper, Hamric highlights problems associated with the lack of consistent definitions and suggests the need for a more universal definition of moral distress to guide quantitative research in this area. Further Hamric raises concerns about taking up Western-centric approaches to researching moral distress and the need for a cultural specific lens in moral distress research. For example, in their research with Ugandan nurses, Harrowing and Mill (2010) highlight the importance of the cultural context in shaping experiences of moral distress. Exploration of moral distress in various cultural contexts, guided by clearly explicitly theoretical and conceptual understandings of moral distress, is needed.

## Empirical Research Tensions

From our review and analysis of existing research, and the conceptual and theoretical issues outlined above, we have identified at least four challenges related to undertaking research on moral distress. These include (1) the fact that research on moral distress has been conducted predominantly with nurses in acute care in a North American context; (2) the predominance of quantitative studies of moral distress, particularly using one tool developed in a specific context; (3) the variable and limited attention to relationships among moral distress, moral agency and ethical climate; and (4) the limited attention to interventions or action to address concerns related to moral distress.

Moral distress has been an issue and concern in nursing for over two decades and thus, has been a focus of considerable nursing research (Wilkinson 1988). Most research on moral distress has focused on moral distress in nursing, most often in acute care settings. Some have observed that nurses are in less powerful positions in the health care hierarchy, so it may be that the phenomena of moral distress has emerged as a focus of concern more commonly because nurses are often conceptualized as victims (Hamric 2010; Hamric and Blackhall 2007; Ulrich et al. 2007). Researchers have undertaken a few investigations of moral distress from the perspective of managers, students, physicians, pharmacists and other health care providers in a variety of community and acute care settings (Brazil et al. 2010; Hamric and Blackhall 2007; Kälvemark et al. 2004; Schwenzer and Wang 2006). These investigations suggest that moral distress is experienced by other health care providers and in other settings. In particular, specific situations that give rise to moral distress vary based on position and profession; and the extent and degree of moral distress experienced varies across disciplines. Clearly, there is a need to expand research on moral distress in disciplines other than nursing and/or approach research on moral distress from an interdisciplinary perspective.

In nursing, research on moral distress has focused on quantitatively measuring the extent and nature of moral distress among nurses in acute care settings (Cavaliere et al. 2010; Corley et al. 2001, 2005; Hamric and Blackhall 2007; Pauly et al. 2009) using the original moral distress scale (MDS) developed by Corley. The MDS is underpinned by Jameton’s definition of moral distress that emphasizes institutional constraints and focuses on individual perceptions of clinical situations. The MDS has been used widely in North America to examine perceptions of the degree and extent of moral distress (Corley et al. 2001, 2005). Alternative measures have been used or are being developed. One such measure, called an ethics thermometer (Wocial) was introduced at the symposium. In her article in this issue, Hamric specifically addresses in depth critical issues related to the development of instruments to measure and monitor moral distress. Clarity and further theoretical development as to the role of individual and structural factors in the development of measures of moral distress are needed.

Researchers studying moral distress have paid variable attention to institutional factors. For example, some studies of moral distress have incorporated measures of ethical climate. Perceptions of moral distress have been found to vary with perceptions of the ethical climate, one aspect of the organizational culture (Corley et al. 2005; Pauly et al. 2009). Researchers have argued that positive ethical climates are necessary to support professional nursing practice and resolution of moral distress (McDaniel 1997, 1998; Olson 1995, 2002; Olson and Hooke 1988).

In a series of studies, our research team found that nurses often found it difficult to enact their professional and ethical values as a consequence of constraints within their practice environments (Rodney 1997; Rodney and Street 2004; Rodney et al. 2002; Storch et al. 2002; Varcoe et al. 2004; Varcoe et al. in press). While organizational climates in health care have been implicated in the development of moral distress, much of the research on quality practice environments and workplaces has not included an explicit focus on ethical dimensions of the workplace (Aiken et al. 2000, 2002; Clarke et al. 2001; Duncan et al. 2001; O’Brien-Pallas et al. 1994, 2003; Shamian et al. 2002; Shindal-Rothschild et al. 1996). If researchers are to account for both individual and structural factors, then the relationships among moral distress, moral agency and ethical climate must be theorized more fully. For further discussion of these issues, see Lützén’s article in this special issue.

In summary, current research is plagued by differing conceptualization of moral distress and, as identified at the outset, this variation has led to a situation in which research has variously emphasized individual and structural factors. Recommendations both to strengthen individual abilities to cope with moral distress and to enhance health care environments through strategies aimed at individuals such as ethics education, providing opportunities for ethics debriefing and/or providing ethics resources (Storch et al. 2009a, b) have been identified as research priorities. However, there has been little discussion as to what structural interventions are required. As Hamric highlights in her paper, there has been limited research on interventions and it is not yet clear what kind of interventions should be pursued. We urge that conceptualization of moral distress and clarity as to the theoretical underpinnings of moral distress in relation to individual and structural factors is urgently needed to inform coherent and actionable recommendations for addressing negative consequences of moral distress.

## Limited Engagement with Policy and Politics

Conceptualizing moral distress as both an individual and structural concern brings to the fore the importance of policy and political influences that shape the context of nursing and health care practice. In work on moral distress, there has been relatively limited engagement with the role of policy or political influences that shape institutional constraints. We see this as a critical area for future work and necessary for an agenda for action on moral distress. Participants in our research have highlighted the role of policy in shaping practice and fostering feelings of helplessness and powerlessness in the face of current policy and political environments (Storch et al. 2002; Varcoe et al. 2004; Varcoe et al. in press). Changes to policy and practice can enhance ethical nursing practice and such changes are needed to support positive resolution of moral distress. For example, in our own research, participants frequently highlighted the importance of talking to peers in the resolution of moral distress, yet they found that organizational constraints such as workload and narrow definitions of efficiency mitigate against such opportunities (Rodney et al. 2002; Storch et al. 2002, 2009a; Varcoe 2004). There is a need for translation of existing research and identification of priorities to guide future research that can inform recruitment and retention strategies in health care. How do nursing and other health care leaders understand moral distress? In our experience leaders’ stances toward moral distress range from seeing moral distress as a signal for needed organizational change, to seeing such distress as an unavoidable part of practice that needs to be accepted, to dismissing moral distress as too all encompassing and therefore not useful. Attention to moral distress is relevant to the development of a strong, vibrant and healthy workforce and the prevention of the common negative outcomes associated with moral distress in the workplace. A healthy workforce is essential to ensuring safe competent and ethical care. Investment in provision of resources for resolution of ethical concerns has been argued to be cost effective (Hart 2005).

Work on moral distress is particularly salient to work on quality practice environments and patient safety initiatives. Hamric (2010) suggests that in addressing moral distress, action is needed at three levels: individual provider, unit and organizational levels. Previous research has suggested that the experience of moral distress is impacted by contextual factors such as availability of resources for practice (Kälvemark et al. 2004; Varcoe et al. in press). A relatively unexamined area is that of the impact of structural inequities on the development of moral distress. For example, how do classed, gendered or racialized processes play out organizationally in the development of moral distress among different disciplines and positions within the hierarchy?

## Limited Attention to Ethics Education

Educators in health care frequently identify ethics education as a key competency for practice and educators are often called upon as a resource for practitioners to enhance their ethics competencies. Much of the emphasis in ethics education of nurses and other health care providers in the past has been on the development of moral reasoning and the ability to draw on and apply ethical frameworks for decision making (Husted and Husted 1995). Often the emphasis has been on ethical dilemmas as opposed to navigating the ethical terrain in everyday practice.

Ethics education can provide a place and space to address and strengthen both individual and systemic responses to moral distress. To date, at least two intervention studies have focused on educational interventions as means of reducing and responding to moral distress among practitioners (Beumer 2008; Rogers et al. 2008). Both entry level and continuing education are areas for action. Nurse educators face particular challenges in balancing competing curriculum demands and making ethics education a priority, and face challenges regarding whether ethics education is best addressed through ethics-specific courses, or as integrated throughout the curriculum. The question of how to strengthen ethics education to enable health care providers to better address moral distress has received little attention in research. In this special issue, Austin describes the suffering of nurses and their attempts to navigate the terrain of moral distress. She highlights the tension between individual and institutionalized conceptions of moral distress and furthers conceptualization of moral distress as relational. A relational approach to conceptualizing moral distress suggests the need to blend individual and institutional factors in research and education as well as guide the development of interventions.

In nursing, moral distress has been situated within concerns about power imbalances between providers and hierarchies of power that are deeply embedded in health care systems. In response, educators and researchers have long called for joint ethics education of nurses and physicians as well as other members of the health care team (e.g., Storch and Kenny 2007). This recommendation also suggests the need for research within and across multiple disciplines.

## Conclusion

In this article, we have provided a beginning overview of the concept of moral distress and highlighted key issues related to conceptual and theoretical development, current research, policy, and education. Further definition and development of the concept of moral distress is needed to underpin research on moral distress. In particular, there is a need for strong theoretical approaches that can balance the tension between individual and structural factors that shape experiences of moral distress. Most of the published literature to date is within nursing, measuring the nature and extent of moral distress quantitatively. We seek to expand this agenda through discussion and examination of a range of perspectives on moral distress that ought to inform future research that will guide education, policy and practice. Specific attention is needed to focus on organizational elements and processes that shape experiences of moral distress and awareness of issues related to moral distress among policy makers. Educators must navigate multiple and competing curricular demands. Further, because of the limited research regarding intervention and education, educators must function with limited knowledge of strategies to assist health care providers to prevent and positively resolve moral distress. This is of concern in undergraduate, graduate and continuing education.

Each of the authors of the papers that follow take up and explore the complexities of moral distress in relation to the identified areas for action (policy, research, education and practice). Our intention is that this series of papers might serve to assess the state of the art in moral distress research, education, practice and policy. Each article explores a different aspect of a moral distress agenda through critical analysis of the issues and current state of work on moral distress in education, research, policy and practice. In the final article in this series, we articulate the progress to date, as well as future directions for an agenda on moral distress that encompasses action in the areas of research, education, practice and policy. We see these symposium papers as a beginning of a new era in moral distress research.
